# Integrative Metabolome and Transcriptome Analysis of Flavonoid Biosynthesis Genes in *Broussonetia papyrifera* Leaves From the Perspective of Sex Differentiation

**DOI:** 10.3389/fpls.2022.900030

**Published:** 2022-05-20

**Authors:** Peng Jiao, Li Chaoyang, Zhai Wenhan, Dai Jingyi, Zhao Yunlin, Xu Zhenggang

**Affiliations:** ^1^Hunan Research Center of Engineering Technology for Utilization of Environmental and Resources Plant, Central South University of Forestry and Technology, Changsha, China; ^2^Central South Inventory and Planning Institute of National Forestry and Grassland Administration, Changsha, China; ^3^College of Forestry, Northwest A&F University, Yangling, China

**Keywords:** *Broussonetia papyrifera*, flavonoids, transcription factors, sex differentiation, WGCNA

## Abstract

Flavonoids are important secondary metabolites involved in plant development and environmental responses. Sex differences in flavonoids are common in plants. *Broussonetia papyrifera* is a dioecious plant that is rich in flavonoids. However, few studies have been done on its molecular mechanism, especially sex differences. In the present study, we performed an integrated transcriptomics and metabolomics analysis of the sex differences in the accumulation of flavonoids in *B. papyrifera* leaves at different developmental stages. In general, flavonoids accumulated gradually with developmental time, and the content in female plants was higher than that in male plants. The composition of flavonoids in female and male plants was similar, and 16 kinds of flavonoids accumulated after flowering. Correspondingly, a significant enrichment of differentially expressed genes and metabolites was observed in the flavonoid biosynthesis pathway. WGCNA and qRT-PCR analyses identified several key genes regulating the accumulation of flavonoids, such as those encoding *CHS, CHI* and *DFR*. In addition, 8 TFs were found to regulate flavonoid biosynthesis by promoting the expression of multiple structural genes. These findings provide insight into flavonoid biosynthesis in *B. papyrifera* associated molecular regulation.

## Introduction

Dioecious plants are an important part of terrestrial ecosystems and play a positive role in protecting species diversity and maintaining ecosystem stability. From an evolutionary perspective, dioeciousness increases genetic diversity, allowing plants to exhibit a variety of morphologies and traits (Al-Dossary et al., 2021). On the other hand, environmental stress or human selection can further contribute to differences in plant traits between sexes. Actually, sex differences in metabolites are common in plants. In *Rhamnus alpinus*, male plants have higher concentrations of defense-related compounds (anthraquinones) in the leaves than female plants, thus indicating increased resistance to biotic stress in male plants (Banuelos and Obeso, 2004). In *Populus cathayana*, under normal conditions, the sucrose concentration of male leaves was higher than that of female leaves. Nitrogen or potassium deficiency significantly enhanced leaf sucrose in females, but had less effect on males (Yang et al., 2015; Wu et al., 2021). Until now, most reports have focused on the physiological basis of dioecious plants, but few studies have been done on molecular biology, which is a crucial question. Because changes in physiological traits may be due to a genetic basis or in combination with other factors such as allocation. It is necessary to distinguish temporary changes in stress from stable genetic features.

Flavonoids, as a class of polyphenolic compounds with a C6–C3–C6 double aromatic ring, are important secondary metabolites in plants development. Flavonoids can improve the adaptability of plants to the various terrestrial environments, and participate in plant ecological defense, such as promoting plant growth (Stafford, 1991), resisting diseases (Agati et al., 2012), resisting the damage of ultraviolet radiation (Harborne and Williams, 2000), scavenging oxygen free radicals (Agati et al., 2009, 2011; Lillo et al., 2010; Tattini et al., 2010), driving away foragers, and preventing the invasion of harmful microorganisms (Falcone Ferreyra et al., 2012). Therefore, flavonoids may be a secondary metabolite in response to the environment. In addition to being beneficial to plants themselves, flavonoids have also been developed and utilized for their medicinal effects. More and more evidences suggest that flavonoids and related compounds can provide beneficial effects on cancer and chronic diseases, including cardiovascular disease, type II diabetes and non-alcoholic fatty liver disease (NAFLD), due to their immunomodulatory, anti-inflammatory and antioxidant properties (González-Gallego et al., 2010; Ying et al., 2013; Pisonero-Vaquero et al., 2014). Sex differentiation of flavonoid content has recently been emphasized because it not only reflects the survival strategies of different sexual plants, but also affects the development and utilization. For instances, the content of flavonoids in female *Hippophae rhamnoides* leaves is slightly lower than that of male plants (Lu et al., 2018). Under limited phosphorus conditions, the synthesis of flavonoids and condensed tannins in leaves, as well as the synthesis of phenolic acids in stems and roots in females *P. tremula*, were greater than in males (Randriamanana et al., 2014). In *Ginkgo biloba*, more phenolics were extracted from leaves of male trees than from female trees (Koczka et al., 2015).

*Broussonetia papyrifera* (paper mulberry) is a dioecious plant belonging to the Moraceae family. Once an important source of forage, the distribution of *B. papyrifera* has expanded with human migration (Chang et al., 2015). It is widely distributed in Asia and Pacific countries, and is an important economic plant in most areas (Zheng et al., 2002). In recent year, due to its easily digestible crude fiber and high protein content, *B. papyrifera* is still used as forage to address the shortage of feedstuff. At the same time, the tree has the characteristics of strong germination, fast growth, resistance to salt stress, heavy metals and air pollution, and has been used as a pioneer plant in polluted areas (Li et al., 2012; Zhang et al., 2013; Zhao et al., 2014). Compared with other woody plants, *B. papyrifera* is rich in flavonoids, polyphenols, and fructose (Feng et al., 2019), which may be the reason why *B. papyrifera* has been used as a traditional Chinese medicine for a long history (Feng et al., 2008). For instance, prenylflavone derivatives from *B. papyrifera* have an inhibitory effect on cancer cells (Guo et al., 2013), and polyphenols from *B. papyrifera* can inhibit coronavirus protease (Park et al., 2017). Broussochalcone A, papyriflavonol A, 3′-(3-methylbut-2-enyl)-3′,4′,7-trihydroxyflavane, and broussoflavan A from *B. papyrifera* have been proved to be potent the main protease (Mpro) inhibitors. There are more effective than the two repurposed drugs (lopinavir and darunavir) and may serve as promising anti-COVID-19 drugs (Ghosh et al., 2021). *B. papyrifera* leaves and roots are rich in flavonoids. The high flavonoid content may result from the significant expansion of gene family in the flavonoid synthesis pathway (Peng et al., 2019). On the other hand, previous studies have shown that there are sex differences in the accumulation of flavonoids in *B. papyrifera* (Zhao, 2011), but the molecular mechanism is still unclear. Understanding synthesis mechanism of flavonoids in *B. papyrifera* leaves of different sexes will not only help us to understand the survival strategies of *B. papyrifera* in the wild, but also help us to select superior varieties.

At present, metabolomic and transcriptomic analysis have been widely used to study the response mechanisms of metabolite accumulation under different conditions (Zhang et al., 2021). In our study, we focus on the molecular mechanisms of flavonoid biosynthesis in *B. papyrifera* leaves, especially their sex differences. Our work aims to: (I) elucidate the accumulation of flavonoids in *B. papyrifera* leaves of different sexes, and (II) screen the key genes for the synthesis of *B. papyrifera* flavonoids. The results of this work may provide insight into flavonoid biosynthesis in *B. papyrifera* associated molecular regulation.

## Materials and Methods

### Plant Materials

*Broussonetia papyrifera* leaves were collected from April 2019 to October 2019 on the campus of Central South University of Forestry and Technology (28°6′25.48″ N, 112°59′37.68″ E), China. Three adult female (F) and three adult male (M) *B. papyrifera* were selected as experimental samples. They were divided into three groups, each consisting of a male and a female, and they were grown together. The distance between each group was about 200 meters. The six samples were the same age and well-grown. Subsequently, three typical phenological periods of *B. papyrifera* were selected for sampling. The first was 30 days after flowering (the flowering stage, F30/M30). At this point, the gender could be clearly distinguished, and the leaves were fully expanded. The second was 120 days after flowering (the fruiting stage, F120/M120). It was the peak fruiting period of *B papyrifera*, with green and red fruits accounting for about half. The third was 180 days after flowering (the defoliation stages, F180/M180). At this point, the leaves began to fall (Supplementary Figure S1). Every treatment was repeated three times. These leaves were immediately frozen in liquid nitrogen and stored at −80°C for metabolite extraction, transcriptome sequencing, and qRT-PCR analysis.

### Total Flavonoids Analysis

The content of total flavonoids was determined by the aluminum nitrate chromogenic method (Yang, 2018). In brief, 1.00 g freeze-dried leaf powder mixed with 5 ml 70% (v/v) ethanol, and then extracted at 50°C for 1.5 h. The supernatant was collected by centrifugation at 8,000 r/min for 10 min at room temperature, and then diluted to 25 ml with 70% (v/v) ethanol. The content of total flavonoids was measured by spectrophotometer UV-1200 (Mapada, China) at 510 nm using rutin as the standard substance.

### Flavonoid Identification and Quantification

100 mg of vacuum freeze-dried leaf samples were dissolved in 1.0 ml 70% (v/v) methanol. The supernatant was collected by centrifugation at 10,000 g for 10 min. The test solution was obtained by filtration with a microporous membrane (0.22 μm Pore Size). Flavonoids were analyzed by Metware Biotechnology Co., Ltd. (Wuhan, China) using an LC-ESI-MS/MS System (UPLC, Shim-Pack UFLC SHIMADZU CBM30A System, www.shimadzu.com.cn/; MS, Applied Biosystems 4000 Q TRAP, www.appliedbiosystems.com.cn/). Metabolite quantification was performed using a scheduled multiple reaction monitoring (MRM) method, which has been previously described (Chen et al., 2013). Setting |Log_2_ (Fold Change)| ≥2 and |Log_2_ (Fold Change)| ≤ 0.5, and *P* < 0.05 as thresholds for DAFs.

### RNA-seq Analysis

The total RNA was extracted from frozen leaf samples using an EASYspin Plus Plant RNA Kit by Biomarker Technologies Co., Ltd. (Beijing, China). A total amount of 1 μg RNA per sample was used as input material for the RNA sample preparations. Sequencing libraries were generated using NEBNext^®^Ultra™ RNA Library Prep Kit for Illumina^®^ (NEB, USA) following manufacturer's recommendations and transcriptome sequencing were performed by Biomarker Technologies Co., Ltd. (Beijing, China) using an Illumina Hiseq 2000 platform. Clean data were obtained by removing reads containing adapter, reads containing ploy-N and low quality reads from raw data. Transcriptome assembly was accomplished using Trinity (Grabherr et al., 2011) by default. The assembled transcript was mapped to the clean reads to obtain the mapped reads. Gene expression levels were estimated by RNA-seq by Expectation-Maximization (RSEM) (Li and Dewey, 2011), and fragments per kilobase of transcript per million mapped reads (FPKM) was used for gene/transcript level quantification. Differential expression analysis of two samples was performed using the EBSeq R package. *P* value was adjusted using *q* value. *q* value < 0.005 and |log_2_ (Fold Change) |>1 was set as the threshold for significantly differential expression. Gene functions were annotated based on the following databases: NR (NCBI non-redundant protein sequences), Pfam (Protein family), KOG/COG/eggNOG (Clusters of Orthologous Groups of proteins), Swiss-Prot (A manually annotated and reviewed protein sequence database), KEGG (Kyoto Encyclopedia of Genes and Genomes), GO (Gene Ontology). We used KOBAS (Chen et al., 2011) software to test the statistical enrichment of differential expression genes in the KEGG pathways.

### Co-expression Network Analysis for Construction of Modules

To identify key regulatory genes in the flavonoid biosynthesis pathway, WGCNA was performed using the WGCNA R package (v 1.70). The adjacency matrix between different genes was constructed with a soft power of 26 and a dynamic tree cut procedure (merge cut height = 0.25, min module size = 30) was used to screen similar modules in the hierarchical tree. The module eigengene was defined as the first principal component of a given module, and then used to represent the expression profile of module genes in each sample. The Pearson correlations between the eigengenes of each module and the abundance of flavonoids were performed using OriginPro 2016. Genes associated with flavonoid biosynthesis pathways were identified by KEGG annotation (ko00941, ko00942, ko00943 and ko00944) (Guo et al., 2020). Genes related to flavonoid biosynthesis and TFs in flavonoid biosynthesis highly correlated modules were constructed co-expression networks with edge weights >0.3, and visualized the candidate target genes with Cytoscape software (Version 3.9.0) (Ding et al., 2021). Genes with high degrees in the network were selected as hub genes.

### qRT-PCR Analysis

Total RNA was extracted from leaf samples according to the instructions of Biofit kit (Tsingke, China). The strand cDNA was synthesized using Goldenstar RT6 cDNA synthesis kit (Tsingke, China). The expression levels of representative unigenes and selected key genes in the flavonoid biosynthesis pathway were analyzed by qRT-PCR using FQD-96A (Bioer Technology, China). The 20 μl reaction mixture contained 10 μl SYBR^®^ Green Real-time PCR Master Mix (CWBIO), 1 μl cDNA template, 0.8 μM of each forward and reverse primer, and 7.4 μl ddH_2_O. The following cycling parameters were applied for amplification: 95°C for 1 min followed by 40 cycles of 95°C for 15 s, 60°C for 15 s, 72°C for 30 s, then followed by 95°C for 5 s, 60°C for 1 min, 0.11°C / s to 95°C, 50°C for 30 s for plate reading. The primers list is shown in Supplementary Table S1. The actin gene was selected as an internal reference (Xu et al., 2019). Three replicates were performed for each sample. Quantitative data were analyzed using the 2^−Δ*ΔCT*^ method.

### Statistical Analysis

Differences between samples were determined by one-way analysis of variance (ANOVA) and significant differences were calculated by the least significant difference (LSD) test at *P* < 0.05.

## Results

### Total Flavonoids in *Broussonetia papyrifera* Leaves

**Figure 1:**
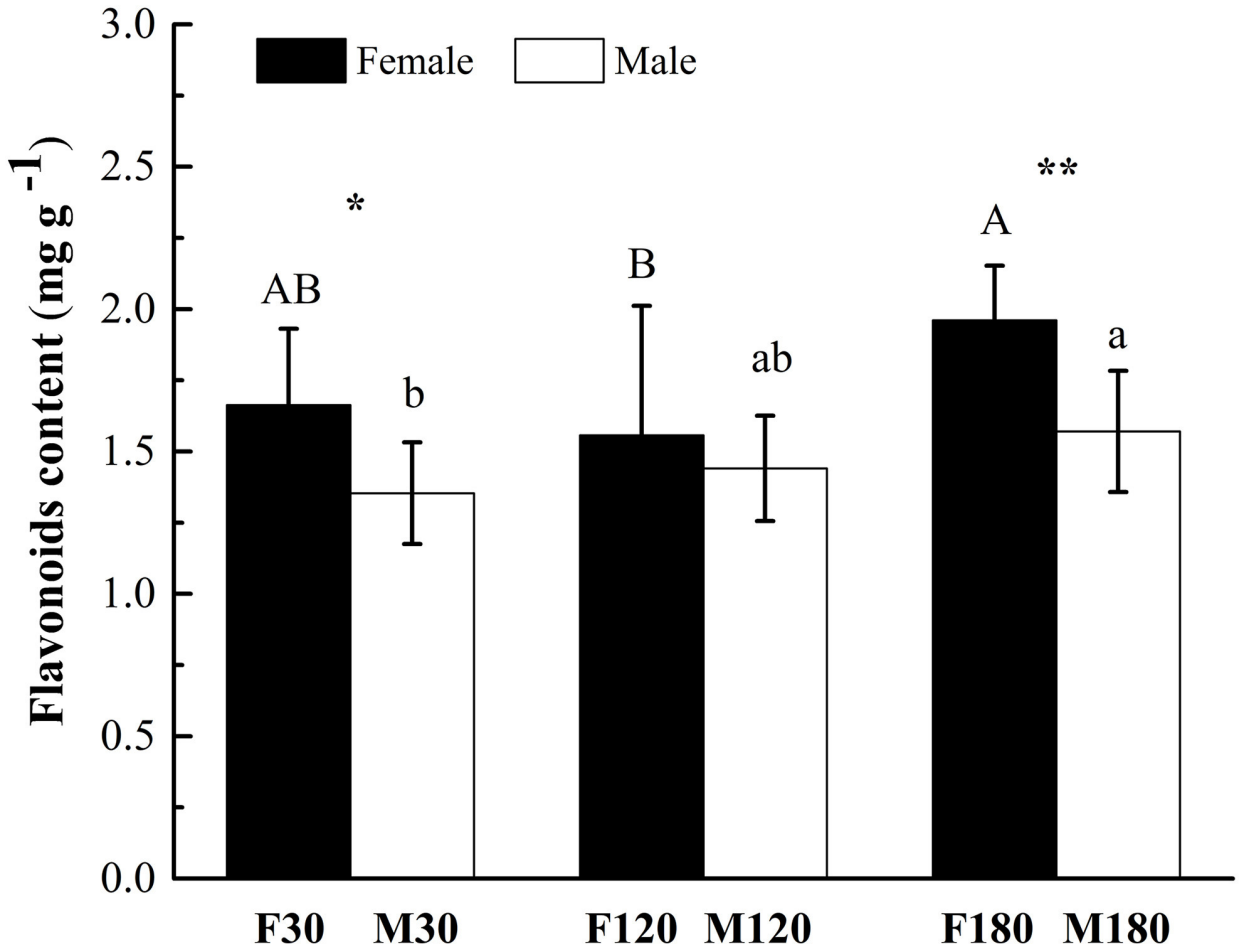
Total flavonoid contents in developing *Broussonetia papyrifera* leaves. Asterisks indicate statistically significant differences between female and male plants: no symbol *P* > 0.05; *P < 0.05; **P < 0.01. Capital letters indicate statistically significant differences (*P* < 0.05) among the three development stages of female plants. Lowercase letters indicate statistically significant differences (*P* < 0.05) among the three development stages of male plants.

The content of flavonoids in leaves of female *B. papyrifera* was higher than that of male at three developmental stages. There were significant differences between flowering and defoliation stages (*P* < 0.05). Accumulation of flavonoids increased with leaf development. F180 and M180 had the highest content at 1.961 ± 0.191 mg g^−1^ and 1.571 ± 0.213 mg g^−1^, respectively (Figure 1).

### Identification of Flavonoids in *Broussonetia papyrifera* Leaves

**Figure 2:**
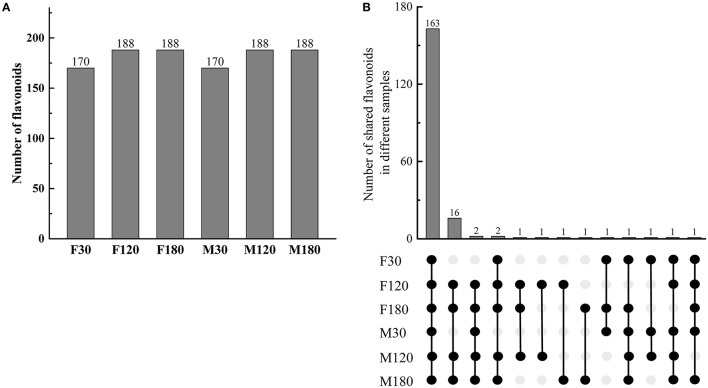
Gender differences in the types and numbers of flavonoid metabolites in *Broussonetia papyrifera* leaves. **(A)** Numbers of flavonoids in different samples. **(B)** Numbers of shared flavonoids in different samples. The black solid circle indicated the presence of compounds in samples, and the gray solid circle indicated the absence of compounds in samples.

**Figure 3:**
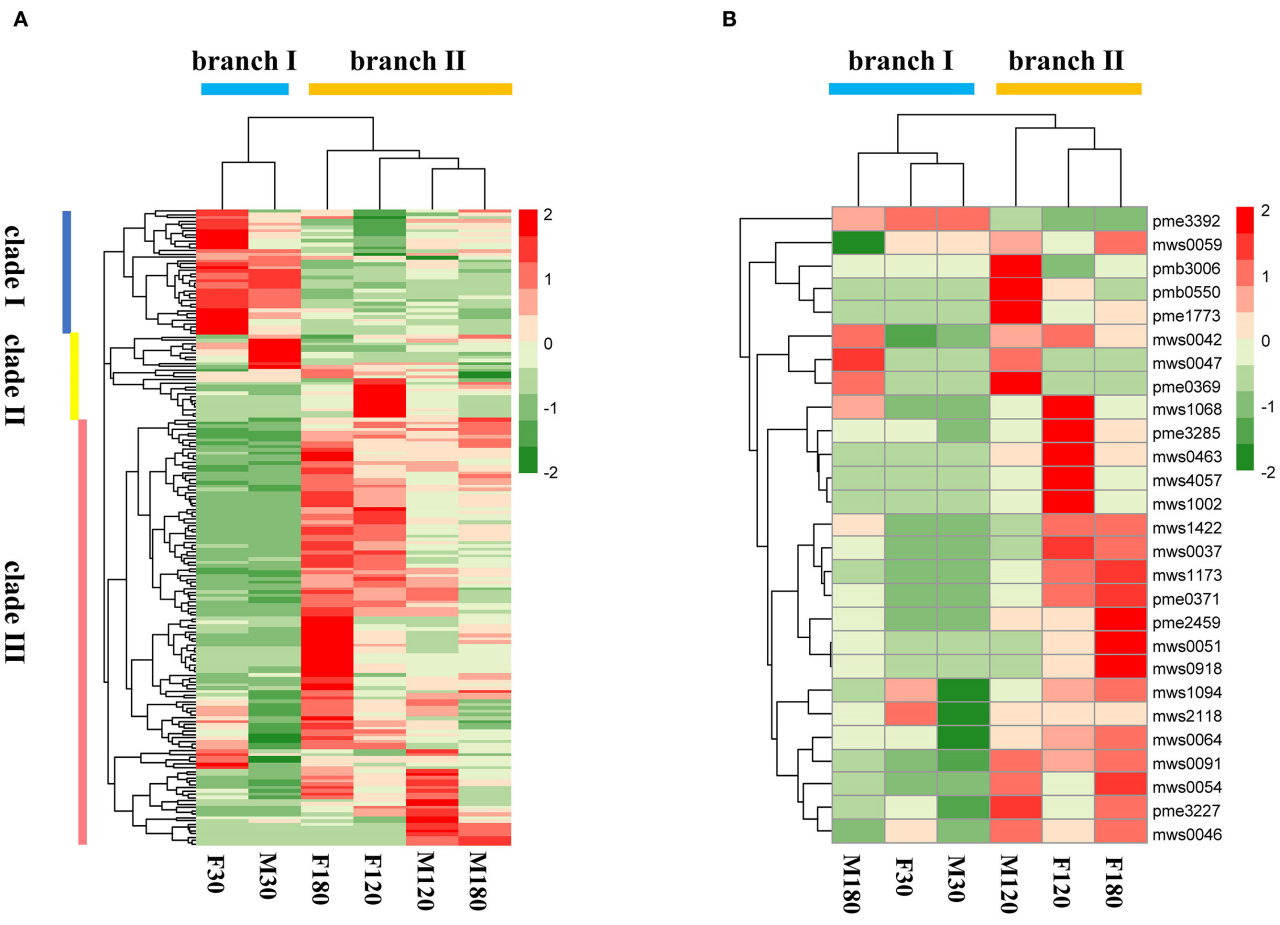
Cluster analysis of flavonoids in *Broussonetia papyrifera* leaves. **(A)** 192 flavonoids found in samples. **(B)** 27 flavonoids identified in the flavonoids biosynthesis pathway.

A total of 192 kinds of flavonoids substances were detected in *B. papyrifera* leaves, including 85 flavones, 46 flavonols, 23 flavonoid carbonoside, 10 flavan-3-ols, 9 anthocyanins, 6 dihydroflavone, 7 dihydroflavonol, 4 isoflavones and 2 chalcones (Supplementary Table S2). 170 kinds of flavonoids were identified in F30 or M30, and 188 kinds of flavonoids were identified in F120, M120, F180 or M180 (Figure 2A). 163 kinds of flavonoids were shared among 6 leaf samples. Besides, 16 kinds of flavonoids were found in the fruiting and defoliation stage but not in flowering stage (Figure 2B). Comparing the sex differences of each flavonoid, there were 50 DAFs in flowering stage, 49 DAFs in fruiting stage and 72 DAFs in defoliation stage. Among this DAFs, the content of most flavonoids in female *B. papyrifera* was higher than that in male (Supplementary Figure S2). Cluster analysis of 192 kinds of flavonoids showed F30 and M30 were located in the same branch, and F120, M120, F180 and M180 were located in the same branch. At the same time, these flavonoids were divided into 3 clade, clade I had high content substances in the F30 and M30, clade II high content substances in the M30 and F120, and clade III high content substances in the F180 and M120 (Figure 3A).

According to KEGG annotation results, 27 substances were identified in the flavonoids biosynthesis pathway. Remarkably, most substances were down-regulated in male samples compared with female samples (Supplementary Table S3). Cluster analysis of 27 flavonoids showed that F30, M30 and M180 were located in the same branch, while F120, M120 and F180 were located in the same branch. The number of flavonoids with high content in the former was lower than that in the latter (Figure 3B).

### Transcriptome Analysis of *Broussonetia papyrifera* Leaves of Different Sexes

The throughput and sequencing quality were high enough to warrant further analysis. Each library received 22244561–22999380 clean reads and 17941528–21223800 mapped reads. The mapped ratio was in the range of 83.79–85.73%. Q30 percentages and GC percentages were 93.08–94.45% and 46.70–47.37%, respectively (Supplementary Table S4). During the development of *B. papyrifera* leaves, a total of 5,672 DEGs were obtained, including 1,475 at flowering stage, 4,391 at fruiting stage and 1,030 at defoliation stage (Supplementary Figure S3). Comparing the DEGs with the common databases, 4,887 differentially expressed genes were annotated, accounting for 86.16% (Supplementary Table S5).

**Figure 4:**
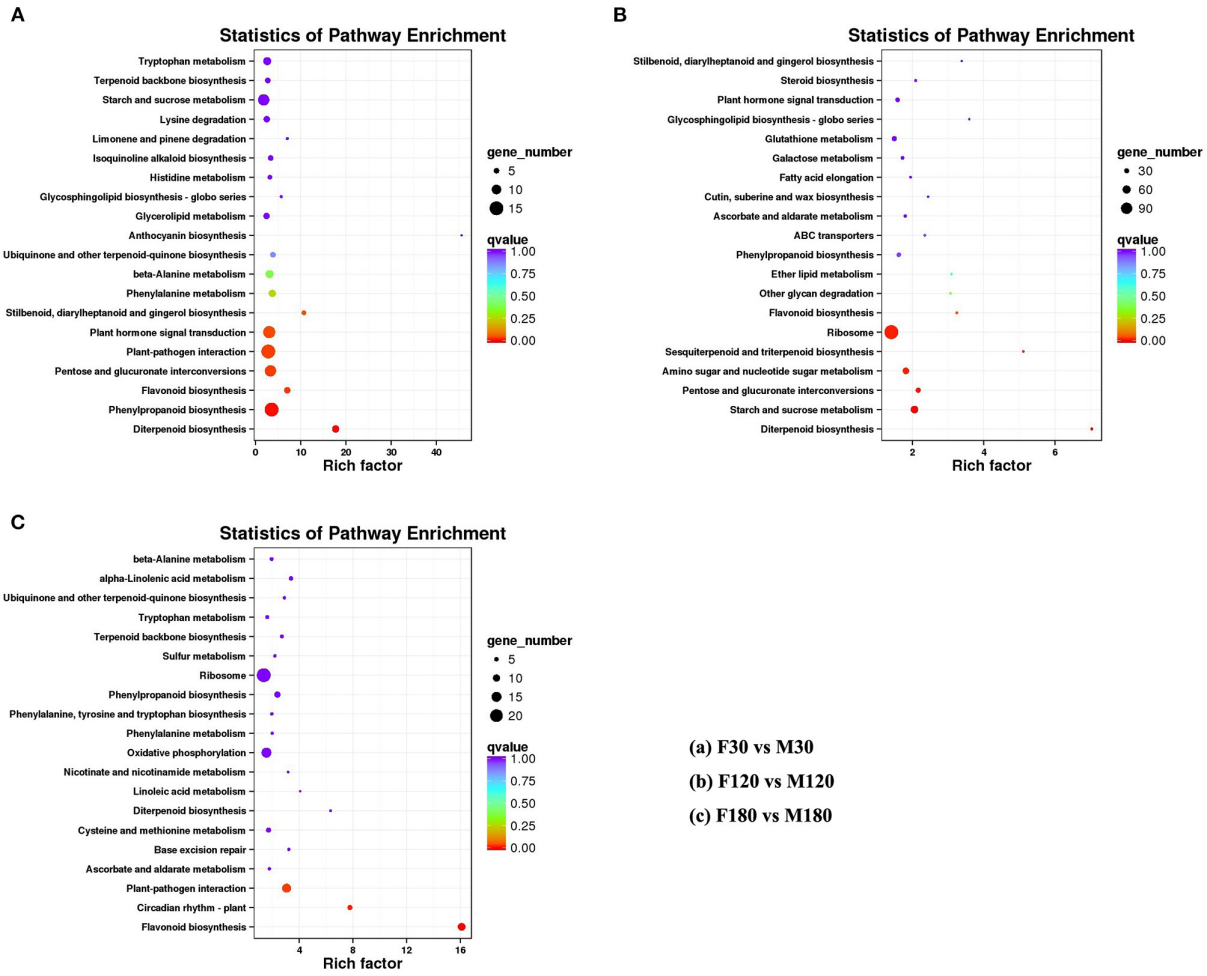
Significant pathways of metabolites in developing *Broussonetia papyrifera* leaves by KEGG functional enrichment. **(A)** F30 vs. M30. **(B)** F120 vs. M120. **(C)** F180 vs. M180.

**Figure 5:**
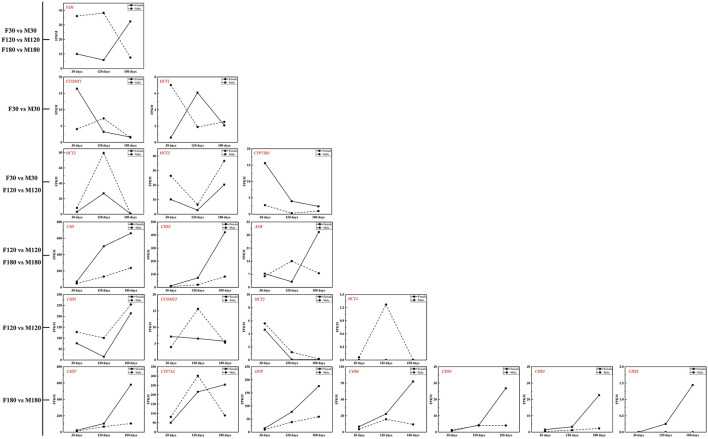
Differentially expressed genes identified in the flavonoids biosynthesis pathway.

The KEGG enrichment analysis revealed that most genes were mainly enriched in “Ribosome,” “Plant-pathogen interaction,” and “Carbon metabolism” (Figure 4). Of note, there were 6 DEGs for F30 vs. M30, 11 DEGs for F120 vs. M120, and 11 DEGs for F180 vs. M180 in the flavonoid biosynthesis pathway. According to KEGG annotation results, 20 genes were identified in the flavonoids biosynthesis pathway (Supplementary Table S6). Among them, 14 genes were only highly expressed in the fruiting and defoliation stages, accounting for 70% (Figure 5; Supplementary Table S6). Among them, *CHI, CHS2, CHS3, CHS4, CHS5, CHS6, CHS7*, and *DFR* were highly expressed mainly in female plants. *CHS1, HCT5* and *HCT4* were highly expressed in male plants. *ANR, CCOMT2* and *CYP73A* were highly expressed in male plants at fruiting stage, and highly expressed in female plants at defoliation stage (Figure 5). Notably, the expression of *F3H* was different in the three stages, the male plants were higher than the female plants in the flowering and fruiting stages, and the male plants were lower than the female plants in defoliation stage (Figure 5). Therefore, some structural genes may regulate the sex differentiation of flavonoid synthesis in *B. papyrifera* leaves, especially in the fruiting and defoliation stages.

### Expression of Flavonoid Synthesis-Related Genes in Different Sexes

**Figure 6:**
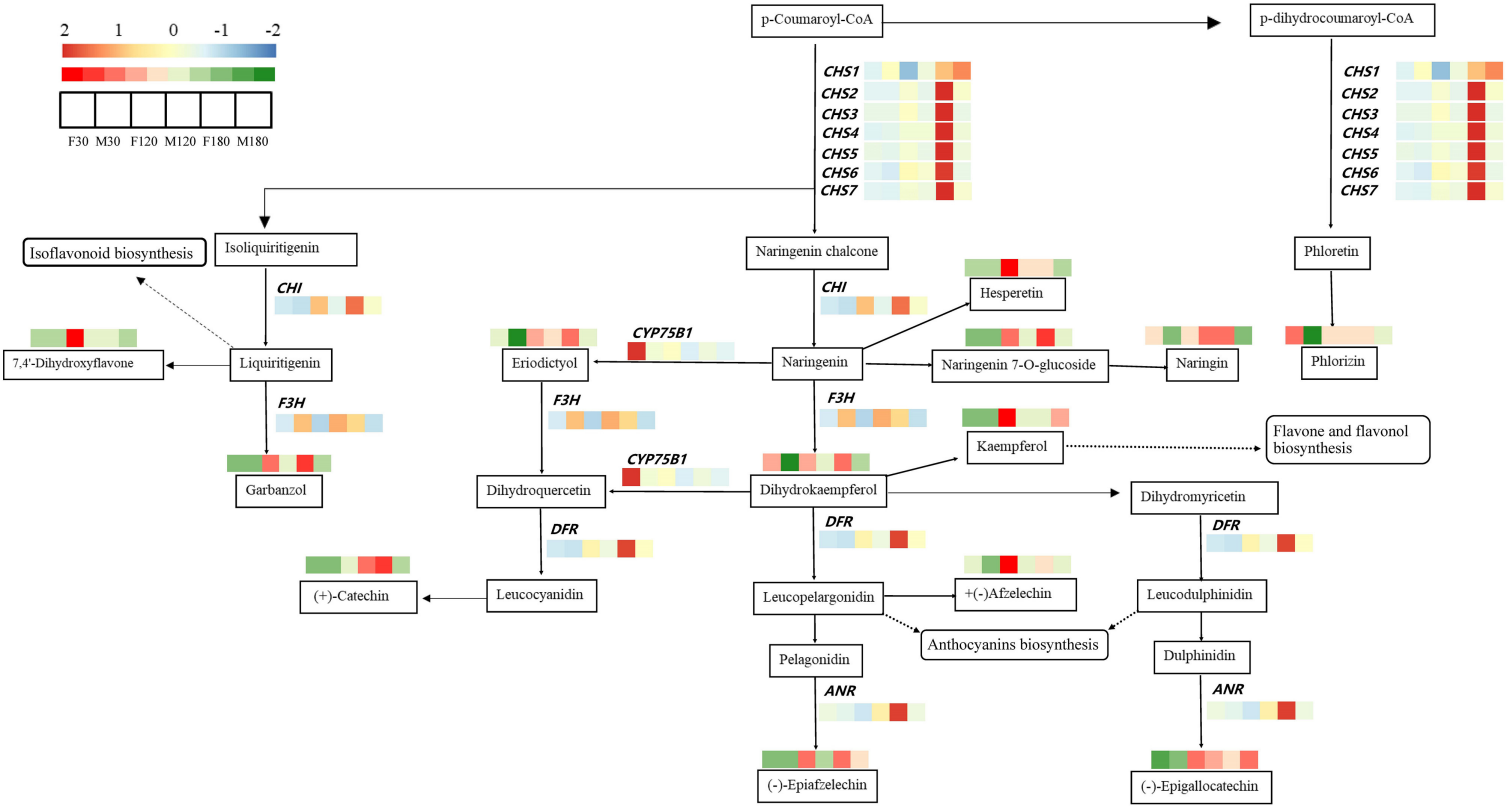
The transcriptional profile of structural genes and metabolites in the flavonoids biosynthesis pathway of *Broussonetia papyrifera* leaves.

The profiles of transcriptional data and metabolic data during leaf development of *B. papyrifera* were exhibited in the flavonoid synthesis pathway (Figure 6). A total of 13 kinds of flavonoids were regulated by 12 genes in the pathway. Among these genes, the differences in gene expression of *F3H, CHS1* and *CYP75B1* had little effect on the content of flavonoids. High expression of *CHS2, CHS3, CHS4, CHS5, CHS6, CHS7, DFR, ANR* and *CHI* promoted the accumulation of flavonoids such as phlorizin, dihydrokaempferol, garbanzol, epigallocatechin, epiafzelechin, catechin, afzelechin, etc.

### Co-expression Analysis to Identify Differential Genes Related to Flavonoid Synthesis

**Figure 7:**
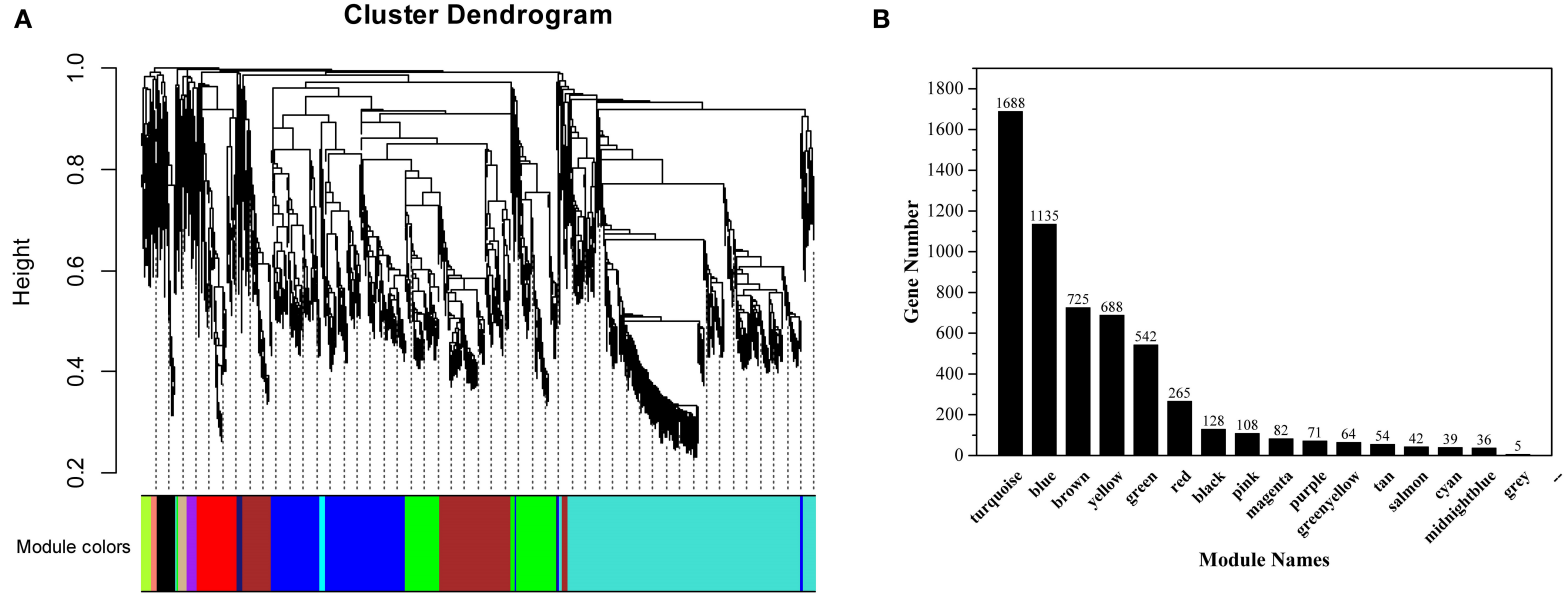
Co-expression network analysis of RNA-seq and physiological trait data. **(A)** Clustering dendrogram of differentially expressed genes and modules identified by the weighted gene co-expression network analysis. **(B)** The number of genes in each module.

A gene cluster tree was constructed according to the correlation of expression levels between genes, and one branch of the tree corresponds to gene clusters whose expression levels were highly correlated (Figure 7A). The average adjacency coefficient of each gene was 196 (Supplementary Figure S4). 16 common expression modules were obtained (Figure 7B). Except for the gray module consisting of non-clustered genes, the turquoise module contained the largest number of genes (1,688). The midnightblue module contained the smallest number of genes (36). Correlation coefficients between modules and flavonoids showed that red and turquoise modules had the highest correlation with flavonoids synthesis (Supplementary Figure S5). The red module was significantly correlated with mws1173 (garbanzol) and pme0371 (naringenin 7-O-glucoside), while turquoise was correlated with mws0463 (hesperetin), mws1068 (kaempferol), mws4057 (7,4'-dihydroxyflavone) and pme3285 (afzelechin) (Supplementary Figure S5).

KEGG enrichment analysis showed that in the red module, flavonoid biosynthesis, cysteine and methionine metabolism, and biosynthesis of amino acids were the top 3 in metabolism pathways (Supplementary Figure S6). Eight genes related to flavonoid synthesis, namely *CHS2, CHS3, CH4, CH5, CHS6, CHS7, ANR*, and *DFR*, were enriched in the flavonoid synthesis pathway (Supplementary Table S6). In the turquoise module, carbon metabolism, biosynthesis of amino acids, oxidative phosphorylation, glycolysis/gluconeogenesis, and purine metabolism were the top 5 in metabolism pathways (Supplementary Figure S6). In addition, a gene related to flavonoid synthesis, *CHI*, was enriched in the flavonoid synthesis pathway (Supplementary Table S6).

**Figure 8:**
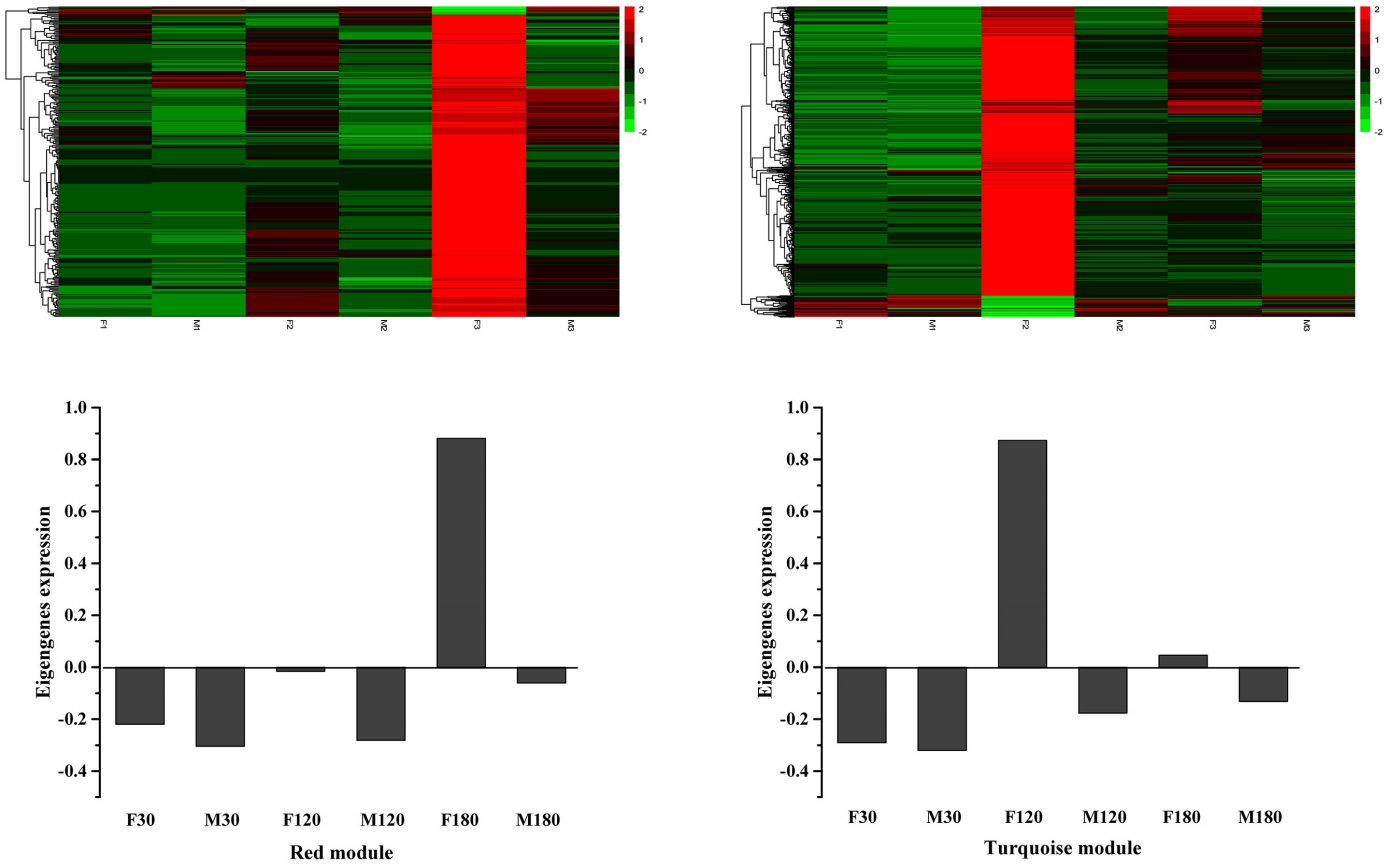
Eigengenes expression profiles of red and turquoise modules.

Using the FPKM values of genes to draw heat maps (Figure 8), the results showed that genes in the red module were highly expressed in F180 and significantly higher than in other samples, while genes in the turquoise module were highly expressed in F120. Although the red and turquoise modules were highly correlated with flavonoid synthesis, the genes in them exhibited distinct expression patterns and might perform distinct functions.

### Identification of Key Genes for Flavonoids Synthesis

Key genes for flavonoids synthesis were screened using the gene expression levels of the major modules (Table 1). In the red module, *CHS2, CH3, CHS4, CHS5, CHS6, CHS7, ANR* and *DFR* were highly expressed at defoliation stage. In the turquoise module, *CHI* were highly expressed at fruiting stage and defoliation stage. *CHS2, CH7, DFR* and *CHI* had FPKM > 100 in our data, these genes may be key genes for flavonoid accumulation of *B. papyrifera* leaves.

**Table 1:** Identification of flavonoid-related genes in the developing *Broussonetia papyrifera* leaves.

**Gene name**	**FPKM values** ^**a**^						**Module**
	**F30**	**M30**	**F120**	**M120**	**F180**	**M180**	
*ANR*	7.34	6.01	3.05	14.08	29.63	7.57	Red
*CHS2*	11.39	6.56	73.33	20.51	**421.1**	82.76	Red
*CHS3*	0	0	0.25	0	1.44	0	Red
*CHS4*	0.79	1.31	4.3	4.07	26.79	4.06	Red
*CHS5*	1.57	0.42	3.25	1.23	22.67	2.28	Red
*CHS6*	10.13	5.48	33.37	23.55	92.84	14.24	Red
*CHS7*	21.69	12.84	**104.69**	67.29	**580.22**	**105.64**	Red
*DFR*	14.67	9.69	77.47	38.38	**175.87**	59	Red
*CHI*	71.88	48.25	**502.77**	**132.94**	**661.16**	**237.24**	Turquoise

a *Expressed unigenes with FPKM values >100 were highlighted with bold font*.

### Identification of Transcription Factors for Flavonoids Synthesis

**Figure 9:**
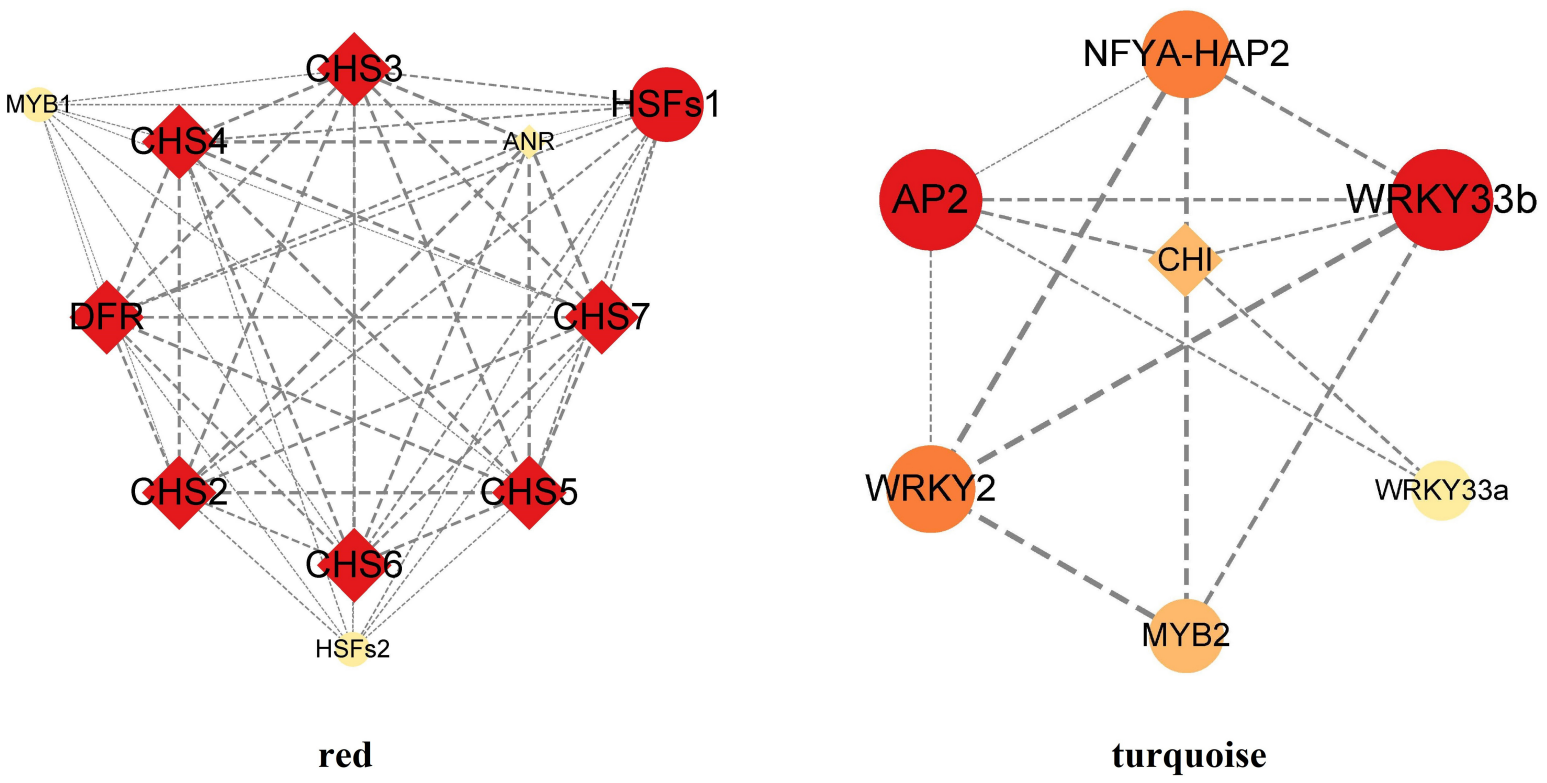
Co-expression network related to flavonoids biosynthesis.

TFs were screened using the co-expression network of the major modules (Supplementary Table S7; Figure 9). The results showed that there were eight structural genes related to flavonoid synthesis, which were *CHS2, CHS3, CH4, CH5, CHS6, CHS7, DFR*, and *CHI*. There were eight TFs related to flavonoid synthesis. Among them, HSFs1, HSFs2, MYB1 were strongly associated with *CHS* and *DFR*. AP2, NFYA-HAP2, WRKY33a, WRKY33b and MYB2 were strongly associated with *CHI*.

### qRT-PCR Analysis

To verify the stability of transcriptome, qRT-PCR verification on 10 representative unigenes in the flavonoid biosynthesis pathway was performed (Supplementary Table S1; Supplementary Figure S7). The correlation coefficient between qRT-PCR results and RNA-seq results reached 0.82. Therefore, our transcriptome sequencing data were generally accurate.

**Figure 10:**
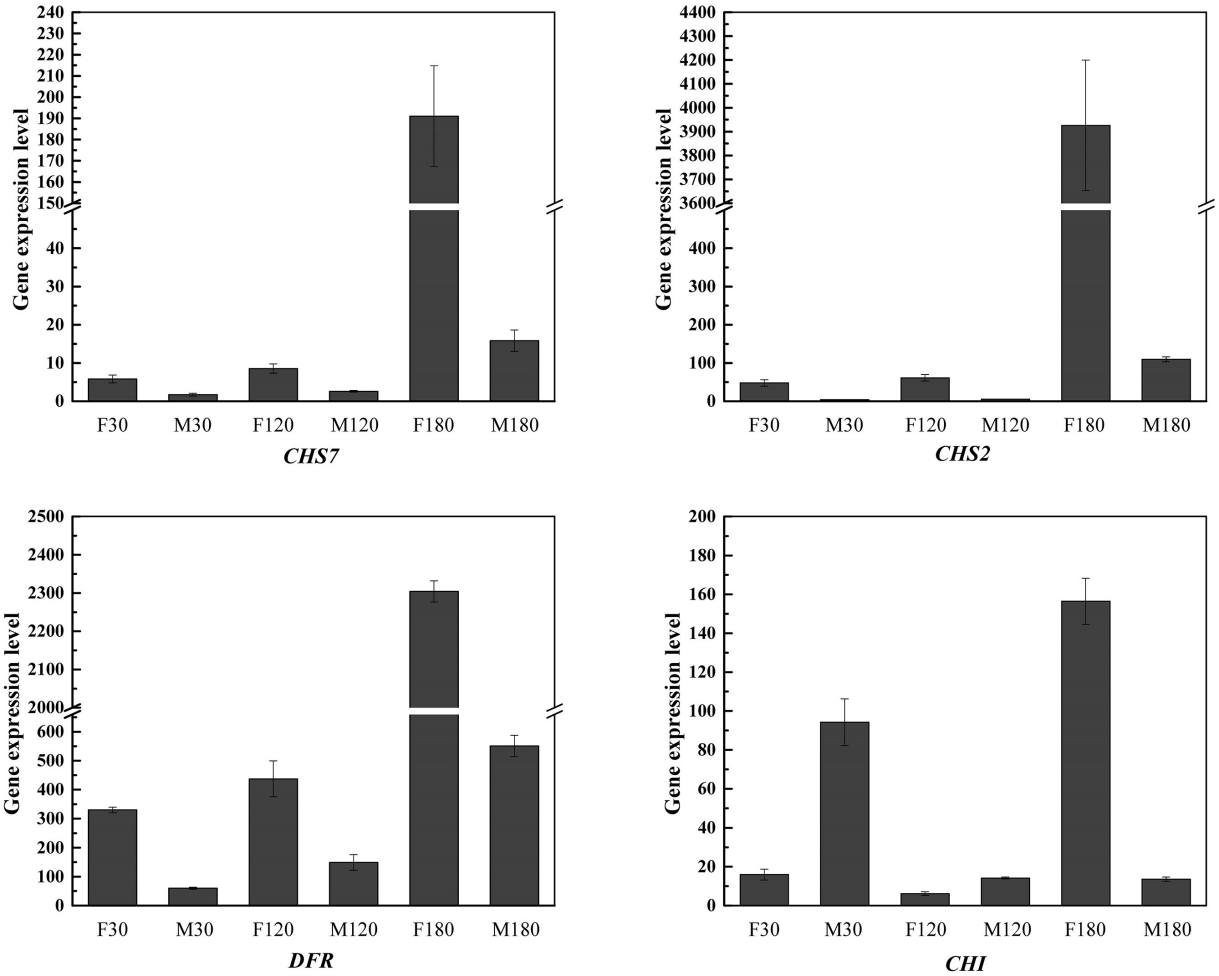
qRT-PCR analysis of four structural genes associated with flavonoids biosynthesis.

On the other hand, qRT-PCR verification on four key genes for flavonoid accumulation of *B. papyrifera* leaves was performed (Figure 10). The results showed that *CHS2, CHS7, DFR* and *CHI* were highly expressed in female plants at defoliation stage. By comparing the differences between female plants and male plants at different stages, *CHS2* gene had the largest difference, reaching 35.77 times.

## Discussion

In the present study, we investigated sex differences in flavonoid accumulation of *B. papyrifera* leaves. The results showed that flavonoids gradually accumulated with developmental time, and the content in female plants was higher than that in male plants (Figure 1; Supplementary Figure S2). Moreover, the composition of flavonoids in female and male plants was very similar, and 16 kinds of flavonoids accumulated after flowering (Figure 2). Xu et al. (2009) studied the seasonal changes of total flavonoids in different parts of *B. papyrifera*, and the results showed that flavonoids in *B. papyrifera* leaves increased with the developmental stage, which was consistent with our research. Generally speaking, the different selection pressures faced by female and male plants lead to different nutritional requirements. Female plants have a higher investment in reproduction and show stronger compensatory mechanisms (Delph and Herlihy, 2012). Studies have found that in resource-rich environments, the sex ratio tends to favor female plants (Ward et al., 2002), while in resource-poor environments, the sex ratio tends to favor male plants (Dawson and Ehleringer, 1993; Chen and Li, 2014; Li et al., 2017). Therefore, we hypothesize that the different survival strategies of male and female plants of *B. papyrifera* leaves lead to differences in flavonoids.

Combined with the results of WGCNA and qRT-PCR analysis, *CHS2, CHS7, CHI* and *DFR* may the key genes for flavonoid accumulation of *B. papyrifera* leaves, and the expression of these genes was affected by sex differences and developmental stages (Figures 5, 9, 10; Table 1). *CHS* directs the phenylpropane metabolic pathway to the synthesis of flavonoids. In *B. papyrifera* leaves, *CHS* enzymes code for proteins exhibiting polyketide synthase activity that accept either p-dihydrocoumaroyl-CoA or p-coumaroyl-CoA as starter CoA substrates, leading to production of phloretin, naringenin chalcone, and isoliquiritigenin (Figure 6), which supports the findings in apple (Yahyaa et al., 2017). *CHI* enzymes (EC 5.5.1.6) accept either naringenin chalcone or isoliquiritigenin as starter, leading to production of naringenin and liquiritigenin (Figure 6). *DFR* enzymes (EC 1.1.1.219) catalyz the NADPH-mediated and stereospecific reduction of dihydroflavonols to leucoanthocyanidins, which are the precursors for the formation of anthocyanins, catechins, and proanthocyanidins (Fischer et al., 2003). Specifically, it accepts either dihydrokaempferol, dihydroquercetin or dihdydromyricerin as starter, leading to production of leucopelargonidin, leucocyanidin and leucodelphinidin (Figure 6). However, these results are based on statistical method, and the function of these genes needs to be further verified. So far, little was known about the flavonoid-regulated genes in *B. papyrifera* leaves. Relevant molecular studies have only demonstrated that four key enzyme families (*CHS, F3'H, I2'H*, and *DFR*) in the flavonoid synthesis pathway exhibited significant expansion (Peng et al., 2014; Feng et al., 2019). The high flavonoid abundance and strong disease resistance of *B. papyrifera* may originate from these expanded gene families.

*CHS2, CHS7, CHI* and *DFR* were highly expressed in female *B. papyrifera* (Figures 5, 10). Most of them are upstream genes in flavonoids biosynthesis pathway. Correlation coefficients between modules and flavonoids showed that *CHS2, CHS7* and *DFR* was significantly correlated with garbanzol and naringenin 7-O-glucoside. *CHI* was significantly correlated with hesperetin, kaempferol, 7,4'-dihydroxyflavone and afzelechin (Supplementary Figure S5). Therefore, we speculate that the key to affecting the accumulation of flavonoids in *B. papyrifera* leaves of different sexes is the expression of upstream genes in flavonoids biosynthesis pathway, and we can increase the accumulation of flavonoids by enhancing the expression of upstream genes.

TFs that may interact with flavonoid genes were screened from two key modules closely related to flavonoids, and 8 reliable TFs were found (Figure 9). Among them, HSFs and MYB TFs were strongly correlated with *CHS*. *DFR*. AP2, NFYA-HAP2, WRKY and MYB TFs were strongly correlated with *CHI*.

The HSFs family mediates the activation of genes responsive to diverse stresses. The N-terminal region of the HSF protein sequence contains a highly conserved DNA-binding domain, which can accurately identify and bind the heat shock cis-elements (HSE) of the downstream target gene promoter (Wang et al., 2020). Our results suggest that HSFs1 and HSFs2 genes in *B. papyrifera* may promote the expression of genes encoding related enzymes, such as *CHS* and *DFR* (Figure 9). The results are consistent with studies on other species. In soybean, HSFB2b can bind to the HSE cis-elements and promotes expression of *GmCHS* and *GmFLS* (Bian et al., 2020). In *Malus domestica*, MdHSFA8a can regulate the expression of *MdDFR, MdFLS*, and *MdANS* (Wang et al., 2020).

The MYB family is one of the largest families of regulatory proteins, and plays important roles in many secondary metabolic pathways (Zhang et al., 2018b). We found that the MYB1 gene was strongly associated with *CHS* and *DFR*, while the MYB2 gene was strongly associated with *CHI* (Figure 9). These genes may be involved in the flavonoid synthesis. In recent years, many MYB genes associated with the flavonoid biosynthesis have been isolated and characterized. Overexpression of GbMYBFL under the control of the *CaMV35S* promoter promoted the accumulation of flavonoids and anthocyanin in *G. biloba* (Zhang et al., 2018b). MYB6, a major regulator of flavonoid synthesis in *P. tomentosa*, up-regulated the expression of flavonoid biosynthetic genes (Wang et al., 2019).

The AP2/ERF TFs can directly or indirectly participate in multiple processes of plant development such as seed development, morphogenesis of organs such as flowers and fruits by responding to the regulation of ethylene, cytokinin and auxin; in addition to primary metabolism. AP2/ERF also has a significant effect on plant secondary metabolism, especially in regulating the synthesis of main medicinal active components (such as artemisinin, paclitaxel and lignin) (Zhang et al., 2018a). In citrus, the *CitCHIL*1 gene is transcriptionally activated by three AP2/ERF TFs, and significantly enhanced the accumulation of flavonoids (Zhao et al., 2021), and our results supported this point (Figure 9).

The WRKY family is involved in plant growth, development, abiotic stress and regulation of secondary metabolites (Dai et al., 2021). Our results suggested that WRKY33a and WRKY33b may regular the transcription of *CHI*, which is involved in the production of naringenin and liquiritigenin in *B. papyrifera*. In addition, the WRKY family is also involved in regulating other pathways of flavonoid synthesis. In apple calli, MdWRKY11 up-regulated the expression of *F3H, FLS, DFR, ANS*, and *UFGT* and increased the accumulation of flavonoids and anthocyanin (Wang et al., 2018). In grape, overexpression of VvWRKY26 possibly promoted the accumulation of proanthocyanidin biosynthesis (Amato et al., 2017).

Although there are few reports on the regulation of flavonoid synthesis by NFYA-HAP2 TFs, the NFYA-HAP2 gene may be involved in the stress resistance *B. papyrifera*. For example, NFYA-HAP2 showed significant responses to heat shock treatment and *F. oxysporum* infection in *Brassica oleracea* (Kim et al., 2015).

At present, based on the statistical method, we have screened out the key genes and TFs that may regulate the synthesis of *B. papyrifera* flavonoids. To provide more detailed information about the function of the genes and TFs in *B. papyrifera*, it is necessary to use biological techniques such as yeast two-hybrid and yeast one-hybrid methods to verify their transcriptional activities and interactions with the target genes promoter in the future study.

## Conclusion

In this study, we presented the integrative metabolome and transcriptome analysis of flavonoid biosynthesis genes in *B. papyrifera* leaves from the perspective of sex differentiation. A total of 192 flavonoids substances were detected in *B. papyrifera* leaves. The accumulation law of flavonoids was that they accumulated gradually with the development time, and the content in female plants was higher than that in male plants. The composition of flavonoids in female and male plants was very similar, and 16 kinds of flavonoids accumulated after flowering. Four key structural genes involved in flavonoids accumulation during leaves development were identified. Most of them are upstream genes in the flavonoids biosynthesis pathway, and overexpression of these genes increased the accumulation of flavonoids, such as garbanzol, naringenin 7-O-glucoside, hesperetin, kaempferol, 7,4'-dihydroxyflavone and afzelechin. Furthermore, 8 reliable TFs (two HSFs, two MYBP, three AP2/ERF, one NFYA-HAP2, and two WRKY) were found. Based on statistical methods and previous findings, these TFs may interact with flavonoid genes *CHI, CHS* and *DFR* in *B. papyrifera*. This study will be useful for further research on flavonoid biosynthesis in *B. papyrifera* associated molecular regulation.

## Data Availability Statement

The original contributions presented in the study are publicly available. The RNA-Seq data presented in the study are deposited in the NCBI's Short Read Archive (SRA) database (http://www.ncbi.nlm.nih.gov/Traces/sra/sra.cgi) under accession number PRJNA819010.

## Author Contributions

PJ: conceptualization, writing—original draft, and visualization. LC: investigation. ZY: supervision and funding acquisition. XZ: methodology and writing review and editing. ZW and DJ: data administration. All authors contributed to the article and approved the submitted version.

## Funding

This work was supported by Key Projects of National Forestry and Grassland Bureau (201801), Postgraduate Scientific Research Innovation Project of Hunan Province (CX20200711), China Postdoctoral Science Foundation (2020M683592), and Natural Science Foundation of Shaanxi Province (2022JQ-202).

## Conflict of Interest

The authors declare that the research was conducted in the absence of any commercial or financial relationships that could be construed as a potential conflict of interest.

## Publisher's Note

All claims expressed in this article are solely those of the authors and do not necessarily represent those of their affiliated organizations, or those of the publisher, the editors and the reviewers. Any product that may be evaluated in this article, or claim that may be made by its manufacturer, is not guaranteed or endorsed by the publisher.

## Supplementary Material

Supplementary Figure S1 Phenotypes of *Broussonetia papyrifera* leaves of different sexes at three developmental stages.

Supplementary Figure S2 Differentially accumulated flavonoids in leaf samples.

Supplementary Figure S3 Differentially expressed genes in leaf samples.

Supplementary Figure S4 Analysis of network topology for various soft-thresholding powers.

Supplementary Figure S5 Module-trait correlations. Each cell contains the corresponding correlation and *p* value. Red is positive correlation. Green is negative correlation.

Supplementary Figure S6 KEGG functional analysis of red and turquoise modules in metabolism pathways.

Supplementary Figure S7 Pearson correlation analysis between qRT-PCR results and RNA-seq results.

Supplementary Table S1 The primers list of genes for qRT-PCR analysis.

Supplementary Table S2 Types and relative contents of flavonoids in *Broussonetia papyrifera* leaves.

Supplementary Table S3 KEGG analysis of differential metabolites.

Supplementary Table S4 Transcriptome sequencing quality analysis.

Supplementary Table S5 Annotation of differentially expressed genes.

Supplementary Table S6 KEGG analysis of structural genes in the flavonoid biosynthesis pathway.

Supplementary Table S7 Candidate co-express genes related to flavonoid biosynthesis.

## Abbreviations

ANR: Anthocyanin reductase
AP2/ERF: APETALA2/ethylene-responsive element binding factors
CHS: Chalcone synthase
CHI: Chalcone isomerase
DAFs Differentially accumulated flavonoids
DFR: Dihydroflavonol reductase
DEGs: Differentially expressed genes
F3H: Flavanone 3-hydroxylase
HSFs: Heat shock factors
MYB: v-myb avian myeloblastosis viral oncogene homolog
NFYA-HAP2: Nuclear transcription factor Y
qRT-PCR: Quantitative reverse transcription-polymerase chain reaction
TFs: Transcription factors
UPLC: Ultra performance liquid chromatography
WGCNA: Weighted gene co-expression network analysis.
