# Supplementary figures and images for: Integrative Metabolome and Transcriptome Analysis of Flavonoid Biosynthesis Genes in Broussonetia papyrifera Leaves From the Perspective of Sex Differentiation
Source: Front Plant Sci. 2022 May 20;13:900030. doi: 10.3389/fpls.2022.900030 (PMC9163962; doi:10.3389/fpls.2022.900030)

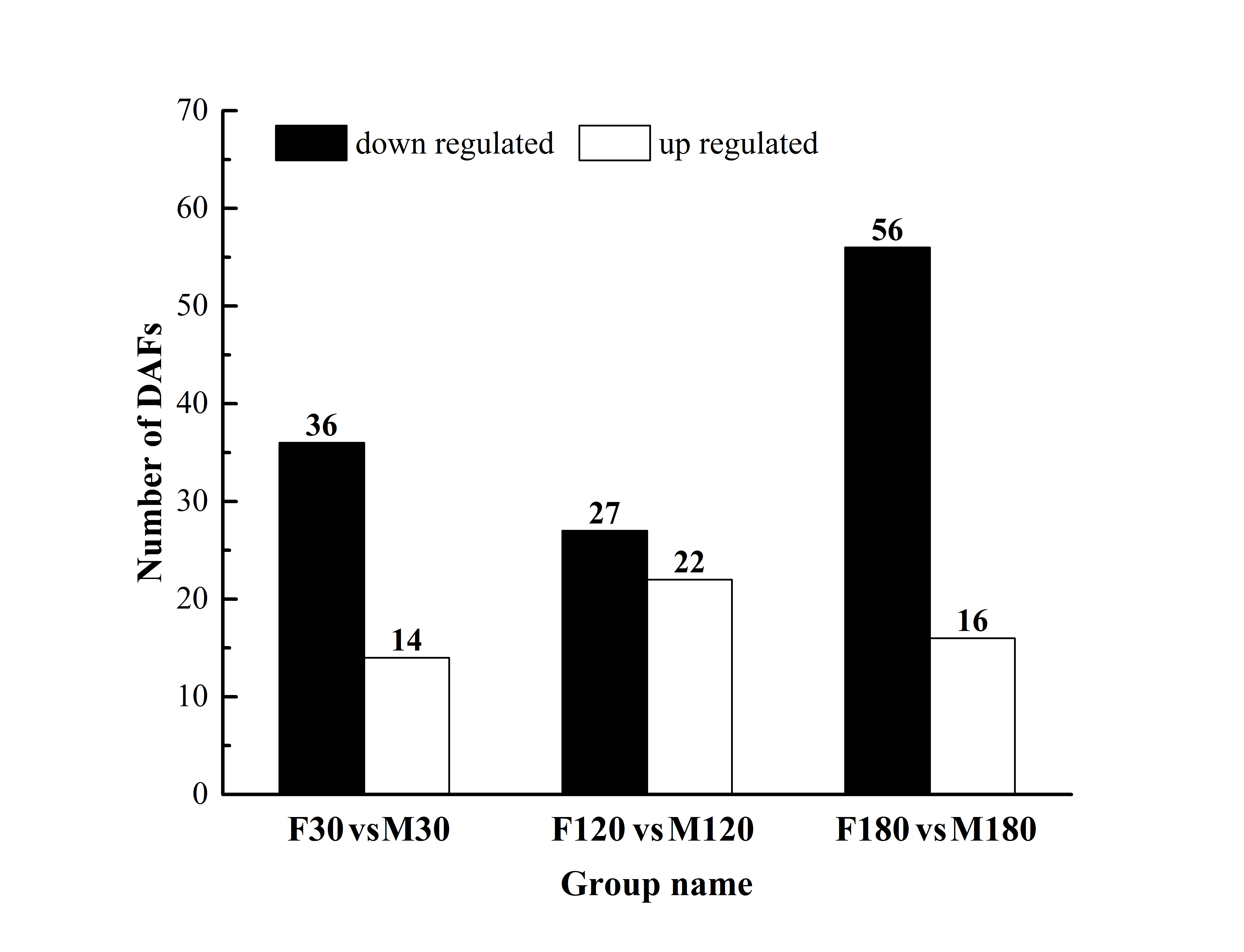

Supplement: Supplementary Figure S2 — Differentially accumulated flavonoids in leaf samples. [file Image_2.JPEG]

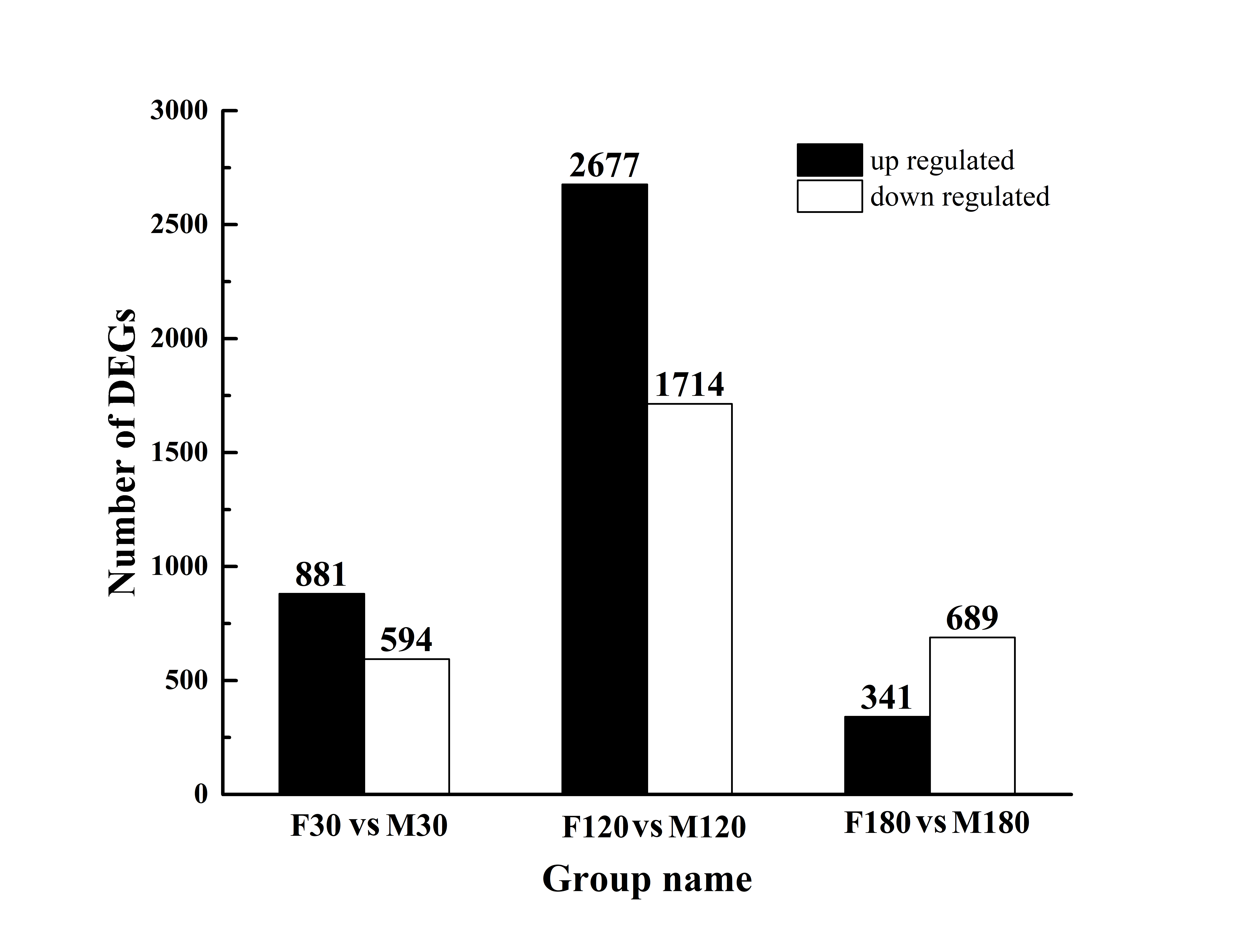

Supplement: Supplementary Figure S3 — Differentially expressed genes in leaf samples. [file Image_3.JPEG]

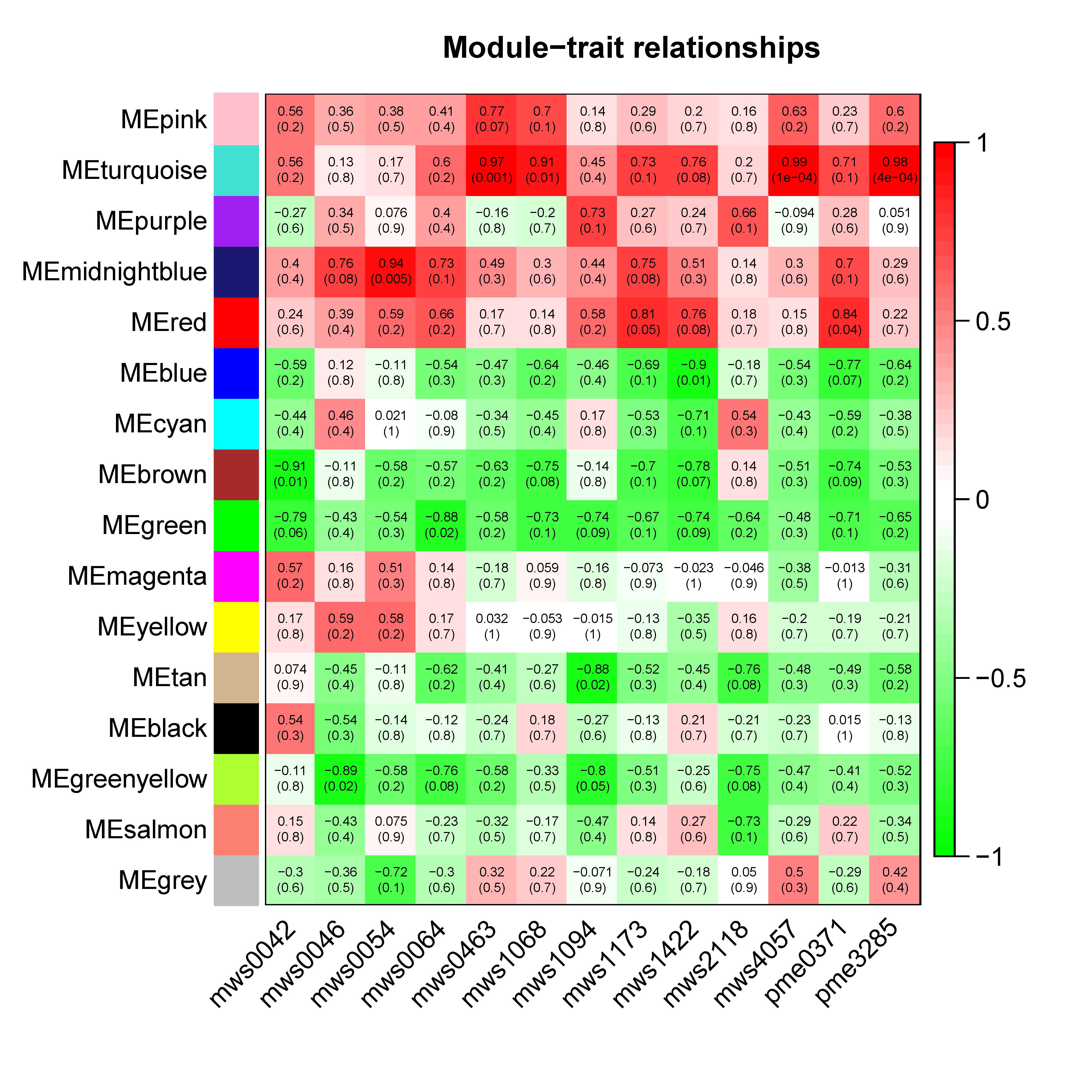

Supplement: Supplementary Figure S5 — Module-trait correlations. Each cell contains the corresponding correlation and p value. Red is positive correlation. Green is negative correlation. [file Image_5.JPEG]

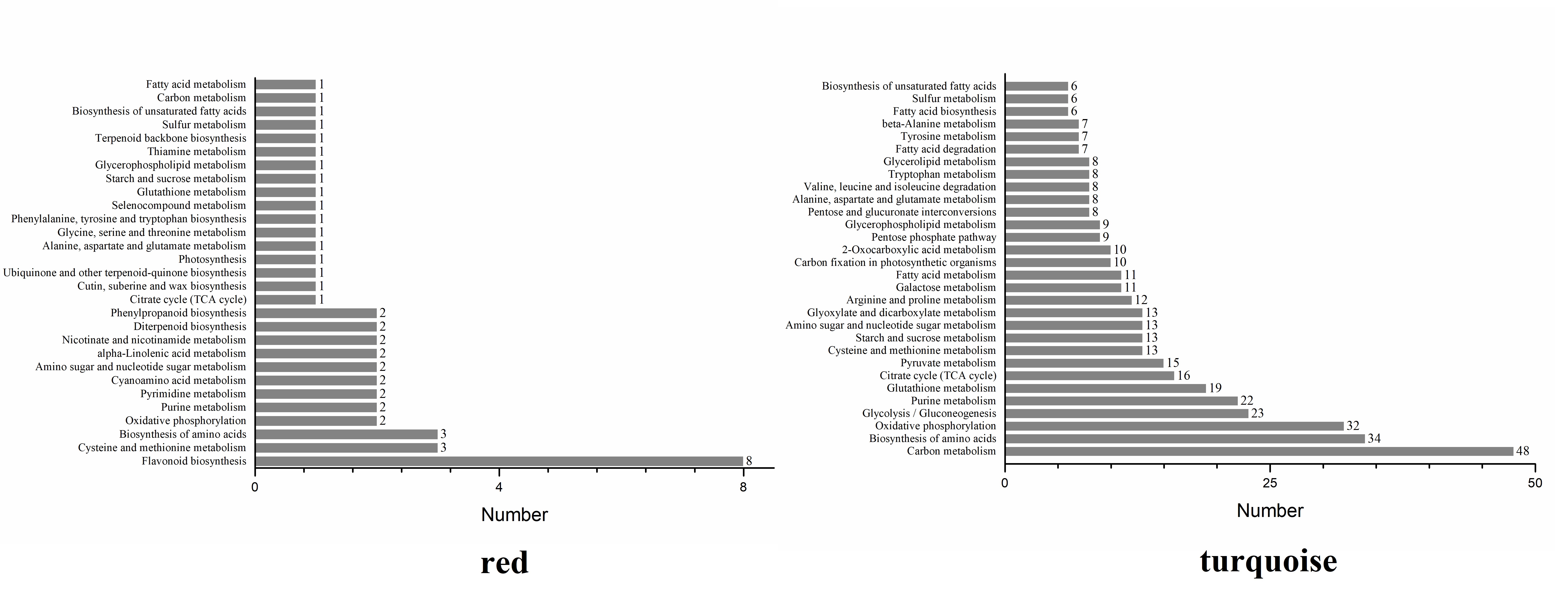

Supplement: Supplementary Figure S6 — KEGG functional analysis of red and turquoise modules in metabolism pathways. [file Image_6.JPEG]

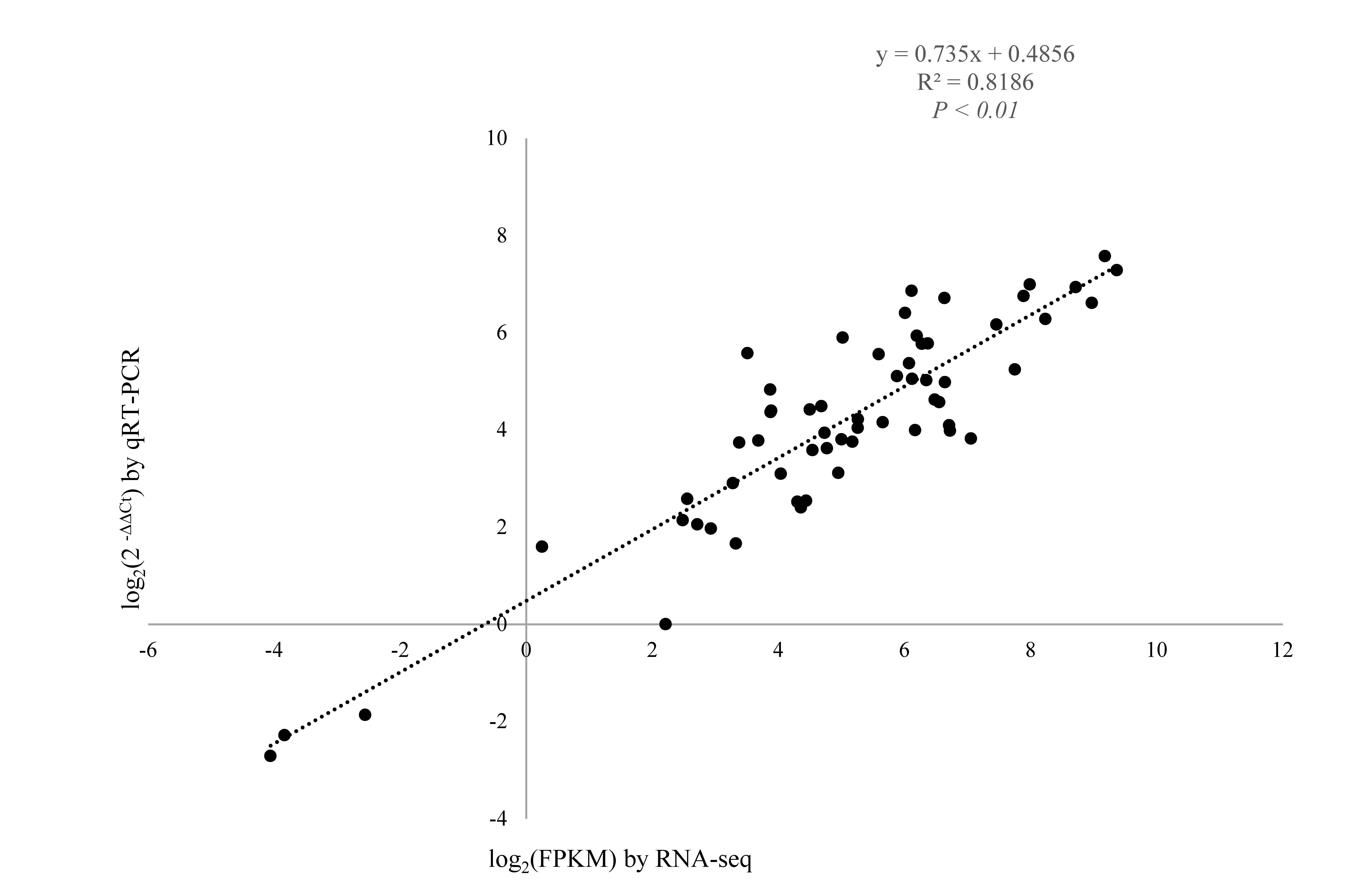

Supplement: Supplementary Figure S7 — Pearson correlation analysis between qRT-PCR results and RNA-seq results. [file Image_7.JPEG]
